# Simultaneous dual targeting of Par-4 and G6PD: a promising new approach in cancer therapy? Quintessence of a literature review on survival requirements of tumor cells

**DOI:** 10.1186/s12935-016-0363-9

**Published:** 2016-11-15

**Authors:** Ingeborg Elisabeth Cernaj

**Affiliations:** Unter der Riede 2, 35274 Kirchhain, Germany

**Keywords:** Cancer, Par-4, G6PD, Targeted therapy, Apoptosis, PI3K/AKT/mTOR, MAPK/ERK, MEK, Aspirin

## Abstract

The aim of this hypothesis is to propose a new approach in targeted therapy of cancer: The simultaneous, dual targeting of two single molecules, Par-4 and G6PD, rather than inhibition of full-length signaling pathways. Rationale: Targeted inhibition of especially two survival signaling pathways (PI3K/AKT/mTOR and MAPK/ERK) is frequently tried, however, a major breakthrough has not yet been reported. Inhibition of complete pathways naturally goes along with a variety of dose-limiting side effects thus contributing to poor efficacy of the administered drugs. This essay offers a synopsis of relevant studies to support the above mentioned idea—targeting of two single molecules which either are crucial for tumor growth and cancer-cell-survival: on one side, Par-4-activation selectively triggers apoptosis of tumor cells thus reversing their characteristic feature—immortality. On the other side inhibition of G6PD breaks the energy supply of tumor cells, weakens their defence against oxidative stress and thereby enhances the sensitivity of tumor cells to oxidative agents (e.g. chemotherapy). Advantage of the proposed dual Par-4/G6PD-therapy is good tolerability and—especially when administered along with conventional therapy—less frequent emergence of resistance.

## Background

Targeted inhibition of especially two survival signaling pathways (PI3K/AKT/mTOR and MAPK/ERK) is frequently tried, however, a major breakthrough has not yet been reported. Inhibition of complete pathways naturally goes along with a variety of dose-limiting side effects thus contributing to poor efficacy of the administered drugs. There is a good case to believe that modulation of single molecules which are crucial for the survival of tumor cells might be more successful.

## Hypothesis

This manuscript deals with the assumption that two well-known molecules—glucose-6-phosphate dehydrogenase (G6PD) and prostate apoptosis response-4 (Par-4)—are some kind of physiological antagonists: G6PD is vital for cell survival whereas Par-4, on the contrary, is required for programmed cell death, apoptosis.

The idea arose that inhibition of the one (G6PD) and strenghtening of the other (Par-4) could be helpful in cancer therapy.

## Supportive evidence

### G6PD strengthens the oxidative defence of tumor cells

Dramatically increased activity of G6PD in cancer cells when compared with the nontransformed type was reported as early as in the middle of the past century [1–7]. This fact has repeatedly been confirmed in more recent studies [8–14] indicating that G6PD plays an important role in the metabolism of cancer cells.

G6PD—the rate limiting step of the pentose phosphate pathway (PPP)—is one of the endpoints of the mTOR-pathway [8, 15, 16] and is therefore regulated by the PI3K/Akt/mTOR-signaling. The activity of G6PD ensures steady supply of pentoses required for the synthesis of nucleic acids and, even more important, for stabilization of the NADP/NADPH-equilibrium which is crucial for antioxidative defence [17]. Both supply with NADPH and with pentoses is an essential prerequisite for the uncontrolled growth and proliferation of cells in general and particularly of tumor cells [8, 15, 18].

### Prostate apoptosis response-4 (Par-4)

Likewise, another molecule plays a central role in tumor development and growth: the tumor suppressor Par-4. Evidence is given that Par-4, which was identified in 1994 in prostate cancer cells [19], plays a key function in apoptosis (for review see [20]).

One of the characteristic features of cancer cells—immortality—is based on deactivation of the Par-4-function to enable the tumor cells to escape apoptosis. Therefore, downregulation of Par-4-expression seems to be a decisive step in tumorigenesis which is vital for the viability of tumor cells [21, 22].

Over the years vast quantities of results dealing with the relevance of the two molecules—G6PD and Par-4—in tumor growth were published. This hypothesis is based on the results gained from search in relevant scientific literature.

Beginning in the late 1970-ies data regarding glucose-6-phosphate dehydrogenase (G6PD)—especially those relating to cell proliferation, oxidative defence and tumor growth—were recorded and analyzed. Research was initially carried out in university- and other scientific libraries. Since online access exists search was continued in large scientific databases like PubMed.

After discovering of prostate apoptosis response-4 (Par-4) by the end of 1990 data regarding this molecule were recorded and analyzed, too, and interpreted in the context of knowledge about the role of G6PD in normal cells as well as in tumor ones.

Research, analyse and interpretation of the findings was done by the author itself over an about 40-year period, beginning with elaboration of PhD thesis in 1976 (“Role of G6PD and its isozymes in human organism”) and continued by personal interest and curiosity until today.

Significant reduction of Par-4-activity was documented in almost all examined tumor-types, among others in kidney- [23], different neurological [24, 25], endometrial [26], breast- [27], prostate- [28], colon- [29] as well as in cholangiocarcinoma-cells [30] thus confirming that reduced Par-4-activity is an important feature of tumor cells.

**Fig. 1:**
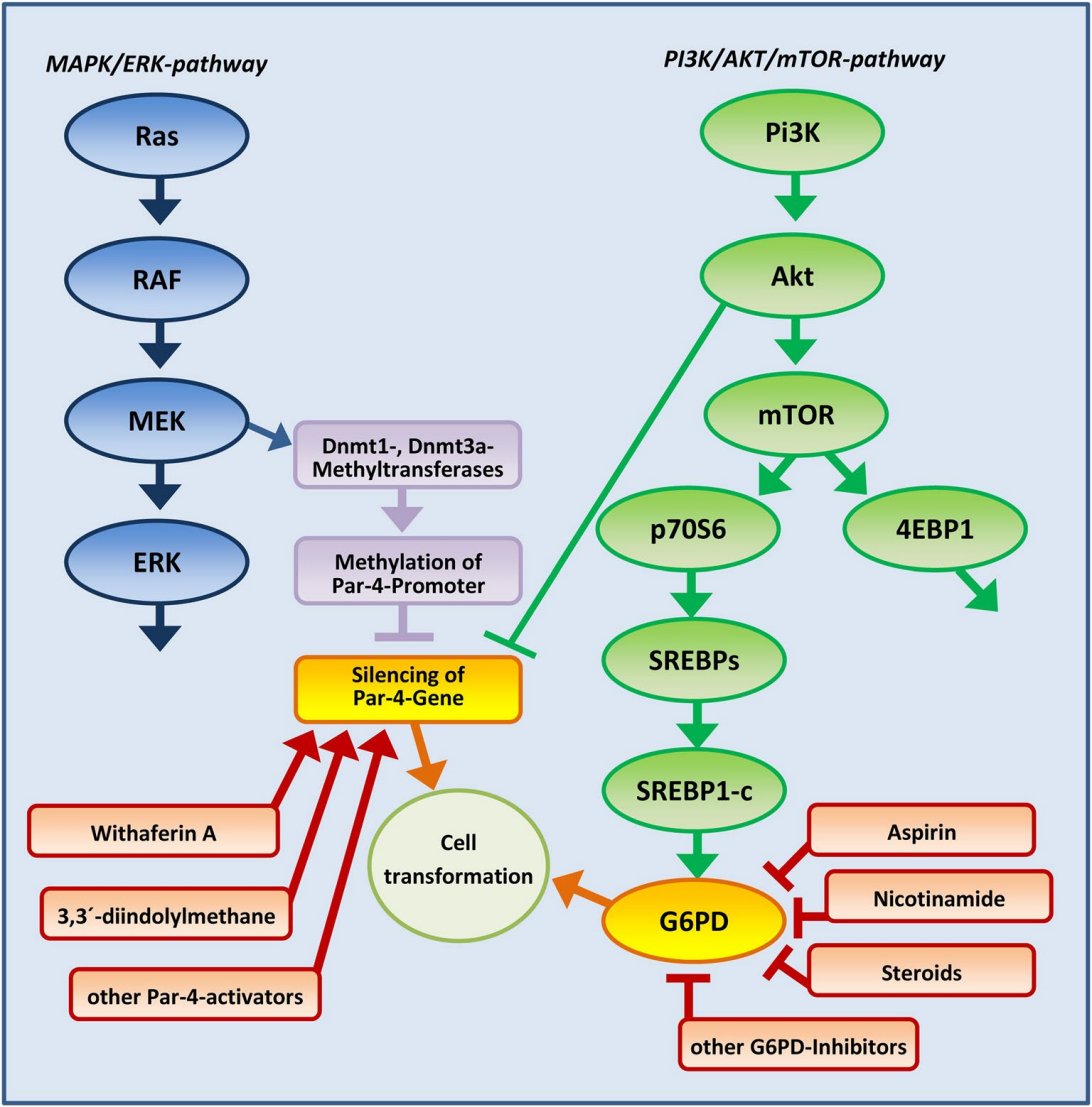
Molecular links between the two major survival pathways MAPK/ERK and PI3K/AKT/mTOR. The vast majority of tumors develop because of oncogenic mutations in the PI3K, Akt, PTEN, ras and related key genes, which are directly involved in initiating of PI3K/Akt/mTOR and/or MAPK/ERK signaling pathways. These pathways are vital for fast growing cells and cell proliferation. In tumor cells, MAPK/ERK and PI3K/AKT/mTOR pathways cooperate in downregulating of the pro-apoptotic tumor suppressor Par-4. Activation of MEK via ras (MAPK/ERK-pathway) induces upregulation of DNA-methylases (Dnmt-1 and Dnmt-3) which methylate specific sites in the promoter of the Par-4 gene hereby silencing the Par-4-gene. On the other hand, activated Akt, a key member of PI3K/Akt/mTOR-signaling, phosphorylates the Par-4 molecule at serine residue 249 hereby triggering downregulation of the Par-4 activity. Deactivation of the Par-4-function enables tumor cells to escape apoptosis and thus ensures their longevity. At the same time, activated PI3K/Akt/mTOR-signaling regulates the activity of G6PD via SREBP1-c. Activated G6PD supplies tumor cells with pentoses for the synthesis of nucleic acids and, even more important, ensures NADP/NADPH-equilibrium, which is vital for antioxidative defense. Withaferin A and 3,3′-diindolylmethane are known Par-4-activators. Aspirin, nicotinamide, steroids are inhibitors of G6PD

Evidence is given that this feature is—one could say—programmed straight from the first step of carcinogenesis. The vast majority of tumors develop because of oncogenic mutations in the PI3K, Akt, PTEN, [31–35], ras [36–39], and other key genes [21, 31, 40, 41]. These genes are—among others—directly involved in initiating of PI3K/Akt/mTOR and/or MAPK/ERK signaling pathways which are vital for fast growing cells and cell proliferation [35]. Mutations of these genes frequently go along with accidental activation of either survival pathways. Both PI3K/Akt/mTOR and MAPK/ERK pathways act in activated state as Par-4-suppressors (see Fig. 1).

### Downregulation of Par-4 via MAPK/ERK

Activation of MEK via ras (MAPK/ERK-pathway) induces upregulation of DNA-methylases (Dnmt-1 and Dnmt-3) which for their part methylate specific sites in the promoter of the Par-4 gene hereby inactivating Par-4. The causative relation between ras, MAPK/ERK and Par-4 was confirmed: Inhibition of MEK causes downregulation of both the DNA-methylases whereon the function of the Par-4 promoter is restored and the Par-4-activity raises again [42, 43].

### Downregulation of Par-4 via PI3K/Akt/mTOR

Likewise, PI3K/Akt/mTOR-signaling is directly involved in downregulation of Par-4. Activated Akt phosphorylates the Par-4 molecule at serine residue 249 in this manner triggering downregulation of the Par-4 activity [44].

As already mentioned several therapeutic approaches based on inhibition of the PI3K/Akt/mTOR and MAPK/ERK pathways were developed [32], i.e. mTOR-Inhibitor Everolimus [45] and BRAF-Inhibitors Vemurafenib and Dobrafenib [46, 47]. These therapies didn’t fulfill the hopes because of dose-limiting side effects [46, 48, 49] and/or development of resistance to the inhibitor [50–53].

### Restoration of Par-4-acitivity causes tumor reduction

In many cases, tumor reduction after restoring of Par-4-activity was reported. So Chakraborty and colleagues observed that injection of Par-4 via adenovirus into prostate tumor induced apoptosis in cancer cells followed by dramatic reduction in tumor volume [54]. Similar findings were reported after Par-4 transfection in melanomas [55]. Vetterkind and colleagues reported that ectopic expression of Par-4 was sufficient to induce apoptosis in tumor cells of the central nervous system [25]. In animal experiments intravenous injection of recombinant Par-4 was sufficient to inhibit the formation of metastases [56]. Yang et colleagues noticed that in vitro activation of Par-4-expression by small activating RNA (saRNA) induced growth inhibition and apoptosis in tumor cells [42].

Treatment of therapy resistant glioma stem cells with a combination of ectopic Par-4 and Tamoxifen caused inhibition of the Akt- and the ERK-pathways followed by subsequent apoptosis in the tumor stem cells. This success was seen only in combination of the two substances while monotherapy either with Tamoxifen or with Par-4 alone failed [57].

Thus, the proapoptotic protein Par-4 seems to be crucial for apoptosis induction in tumor cells; many (if not the vast majority of) cancers are able to develop only because of inactivity (or reduced activity) of this tumor suppressor.

### Inhibition of G6PD arrests tumor growth

Likewise, a direct connection between tumor growth and G6PD was confirmed. Several researchers consider a high G6PD activity in tumors as an independent negative prognostic marker in cancer [8, 56]. Overexpression of G6PD is considered as a predictor of high risk of recurrent metastasis in breast cancer patients [58].

First evidence that reduction of G6PD activity may be capable to reduce tumor growth was given as early as in the 1970-ies [59]; the same was recently approved by more modern methods [9]. It has been shown that reduction of the G6PD activity in tumor cells up to 80% is sufficient for significant reduction of cell proliferation, migration and invasiveness as well as for significant decrease of colony forming efficiency; coincidentally the rate of tumor cell apoptosis increased [9]. Furthermore, it has been shown that inhibition of G6PD activity triggers the sensitivity of tumor cells against oxidative stress and consequently leads to an increased susceptibility of these cells to apoptosis [9, 60].

### Adult organisms are only marginally dependent on G6PD-activity

In resting cells the G6PD-activity is often barely detectable; only regenerating [61] and embryonic cells [62] and, as above mentioned, tumor cells, are dependent on sufficient G6PD-activity. As early as in 1965 Beaconsfield realized that cancer mortality seemed to be lower in populations where G6PD-deficiency is common due to endemic occurrence of deficient G6PD-variants [63]. The well-known finding that regular use of medications with G6PD-inhibiting properties (e.g. aspirin) goes along with a lower cancer risk when compared with the general population underpins the crucial role of active G6PD in tumor formation and growth [64, 65].

## Discussion and conclusions

Targeted inhibition of the prosurvival, antiapoptotic G6PD-activity combined with simultaneous or consecutive targeted activation of the pro-apoptotic tumor suppressor Par-4 might be a promising approach in cancer therapy. Of particular advantage seems to be the fact that combination of the proposed dual targeted therapy with conventional cancer therapies promises to be more effective than either monotherapy [57] notably because of the additional increase of the sensitivity of tumor cells against chemotherapy because of impairment of antioxidative defence mechanisms [66].

### Targeting of G6PD and Par-4 is well tolerated

Controlled G6PD-inhibition is generally well tolerated: serious adverse effects from G6PD-inhibition—at least for a defined period—are in general scarce to be expected (except in case of pregnancy or regenerating processes). In G6PD-deficient individuals hemolytic anemia possibly can occur which has to be clinically managed. The most suitable Inhibitors of G6PD-activity are aspirin [67, 68] and, yet better tolerated, Niacin (6-Aminonicotinamide) [69].

Likewise, Par-4-activation is well tolerated. The proapoptotic effect of Par-4 is selectively restricted to tumor cells. Even activated Par-4 is not capable to induce apoptosis in normal cells; instead of that cells with active Par-4 are senisitized towards apoptotic signals [54, 70, 71].

Evidence is given from vitro- and in vivo-animal studies that several steroids of plant origin (Withaferin A; 3,3′-diindolylmethane) are known as Par-4-activators and are capable to induce apoptosis and growth arrests in prostate [72], cholangio-[73] and other carcinoma cells [73–78]. These natural occuring compounds are generally well tolerated (e.g. Withaferin A is well known from Ayurveda therapy).

Summary: The special advantage of a dual targeted G6PD- and Par-4-therapy rather than inhibition of complete signaling pathways (e.g. PI3K/Akt/mTOR and/or MAPK/ERK) might be good tolerability, no mentionable adverse effects, and—especially in combination with conventional oncological therapy—less frequent emergence of resistance.

## Abbreviations

Akt: serin/threonin-kinase (proteinkinase B, PKB)
BRAF: proto-oncogene protein B-raf
Dnmt: DNA methyltransferases
ERK: extracellular-signal regulated kinase
G6PD: glucose-6-phosphate dehydrogenase
MAPK: mitogen-activated protein kinase
MEK: MAPK/ERK kinase
mTOR: mechanistic target of rapamycin (also known as mammalian target of Rapamycin)
Par-4: prostate apoptosis response-4, also known as PAWR
PI3K: phosphoinositide 3-kinase
PPP: pentose phosphate pathway, also known as ‘hexose monophosphate shunt’ or ‘phosphogluconate pathway’
PTEN: phosphatase and tensin homolog
ras: proto-oncogene (rat sarcoma)
saRNA: short activating RNA
